# Structural basis for the hydrolytic activity of the transpeptidase-like protein DpaA to detach Braun’s lipoprotein from peptidoglycan

**DOI:** 10.1128/mbio.01379-23

**Published:** 2023-10-13

**Authors:** Hsiu-Jung Wang, Víctor M. Hernández-Rocamora, Chiao-I Kuo, Kan-Yen Hsieh, Szu-Hui Lee, Meng-Ru Ho, Zhijay Tu, Waldemar Vollmer, Chung-I Chang

**Affiliations:** 1 Institute of Biological Chemistry, Academia Sinica, Taipei, Taiwan; 2 Centre for Bacterial Cell Biology, Biosciences Institute, Newcastle University, Newcastle upon Tyne, United Kingdom; 3 Institute for Molecular Bioscience, The University of Queensland, Brisbane, Queensland, Australia; 4 College of Life Science, Institute of Biochemical Sciences, National Taiwan University, Taipei, Taiwan; Columbia University, New York, New York, USA

**Keywords:** DpaA, amidase, Braun's lipoprotein, peptidoglycan

## Abstract

The peptidoglycan layer is a defining characteristic of bacterial cells, providing them with structural support and osmotic protection. In *Escherichia coli*, this layer is linked to the outer membrane via the abundant membrane-anchored protein Lpp, known as Braun’s lipoprotein, with LD-transpeptidases LdtA, LdtB, and LdtC catalyzing the attachment. However, one distinctive member of the YkuD-type transpeptidase family, LdtF (recently renamed DpaA), carries out the opposite reaction of detaching Lpp from the peptidoglycan layer. In this study, we report the crystal structure of DpaA, which reveals the enzyme’s ability to cleave, rather than form, the Lpp-peptidoglycan linkage. Assays with purified peptidoglycan-Lpp as the substrate and chemically synthesized compounds suggest that DpaA’s shallow L-shaped active site can only accommodate and cleave the peptidoglycan-Lpp cross-link with a constrained conformation. This study provides insights into how homologous Ldt enzymes can perform opposing chemical reactions.

**IMPORTANCE:**

Cross-linking reaction of Braun's lipoprotein (Lpp) to peptidoglycan (PG) is catalyzed by some members of the YkuD family of transpeptidases. However, the exact opposite reaction of cleaving the Lpp-PG cross-link is performed by DpaA, which is also a YkuD-like protein. In this work, we determined the crystal structure of DpaA to provide the molecular rationale for the ability of the transpeptidase-like protein to cleave, rather than form, the Lpp-PG linkage. Our findings also revealed the structural features that distinguish the different functional types of the YkuD family enzymes from one another.

## INTRODUCTION

Gram-negative bacteria, such as *Escherichia coli*, have a cell envelope composed of a cytoplasmic membrane, a periplasm containing the peptidoglycan (PG) layer, and an outer membrane (OM) (1). PG is a flexible polymer (2), forming a mesh-like sacculus around the cytoplasmic membrane to protect the cell from environmental stress. PG is connected to the OM through association with many OM proteins, such as OmpA, Pal, and porins (3). In particular, PG forms covalent linkage with Lpp (Braun’s lipoprotein), an abundant OM protein widespread among Gram-negative bacteria (4). Thus, Lpp plays a crucial role in maintaining the structural and functional integrity of the cell envelope (5). Lpp is inserted via its N-terminal lipid anchors into the OM layer and is covalently linked to PG via its C-terminus (6). The periplasmic part of Lpp is made of a single α-helix, and the protein forms trimers (7). About one-third of the Lpp molecules are covalently attached to PG via the ɛ-amino group of the C-terminal lysine, which is linked to the α-carboxylic group at the L-center of meso-diaminopimelic acid (mDAP) at position 3 of a PG stem peptide, catalyzed by the LD-transpeptidases (LD-TPases) LdtA, LdtB, and LdtC (8). Although it is not clear how bacteria regulate the attachment of Lpp to PG, this linkage determines the distance between the OM and PG layers, stabilizes the cell envelope (9), and enables the OM to act as a permeability barrier for various toxic molecules and antibiotics (10).

**Fig 1:**
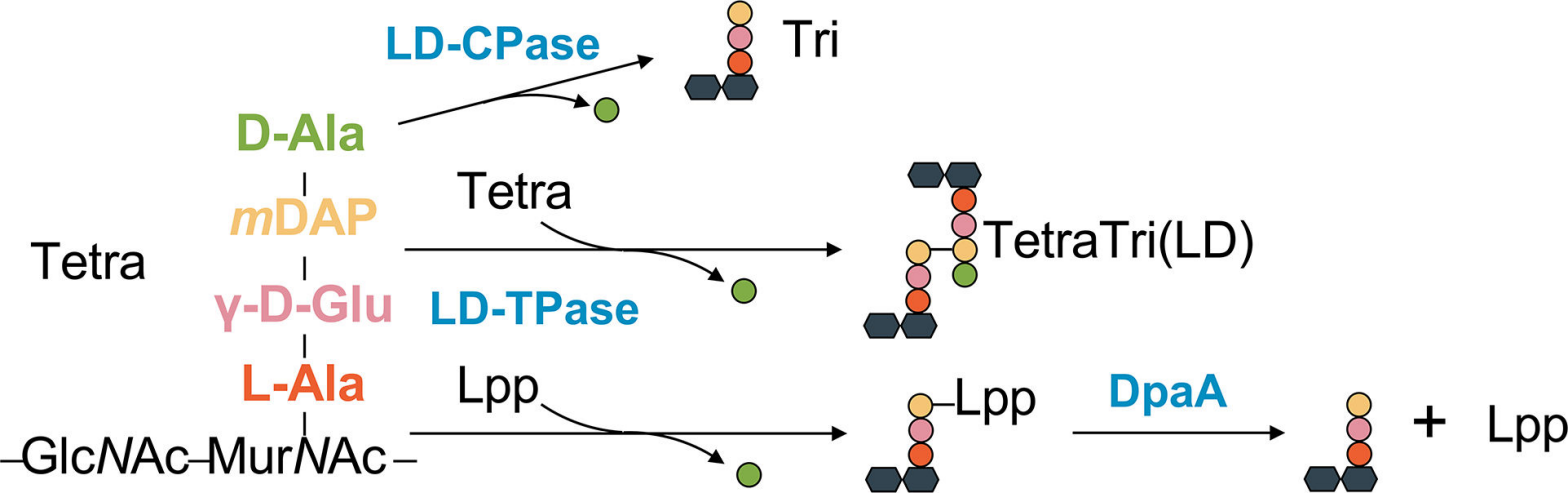
Diverse reactions on peptidoglycan substrates catalyzed by members of the YukD family. The YkuD family includes proteins possessing LD-carboxypeptidase (LD-CPase) or LD-transpeptidase (LD-TPase) activities. DpaA, a unique member of the YkuD family, catalyzes the cleavage of the cross-link between the muropeptide (Tetra) and Braun’s lipoprotein (Lpp).

The Ldt enzymes are members of the YkuD protein family, named after the first identified protein, YkuD, from *Bacillus subtilis* (11). The catalytic domain of this protein forms a β-α-β sandwich structure, wherein a single α-helix is surrounded by two mixed β-sheets. The active site cysteine residue is located on a solvent-exposed catalytic loop in one of the β-sheets. Most proteins in the YkuD family are either LD-TPases or LD-carboxypeptidases (LD-CPases), using PG tetrapeptides with a terminal D-alanine as substrate. Some of the LD-TPases facilitate the formation of the 3-3 cross-links in PG (LdtD, LdtE) (12 – 14), others attach Lpp to PG (LdtA-C) (8) (Fig. 1). LD-CPases of the YkuD family remove the terminal D-Ala from the PG tetrapeptides, for example, Csd6 from *Helicobacter pylori* (15), or Pgp2 from *Campylobacter jejuni* (16), or Bd1075 from *Bdellovibrio bacteriovorus* (17). However, another member of the YkuD family, DpaA (also known as YafK or LdtF), has recently been demonstrated to be an amidase instead of an LD-TPase or LD-CPase (18, 19). DpaA specifically hydrolyzes the amide bond between mDAP in PG and the C-terminal L-lysine of Lpp, in a reaction that is the opposite of the Lpp attachment reactions catalyzed by some of the Ldt enzymes (Fig. 1). These findings suggest a new role for DpaA in regulating PG-OM linkages to resist abnormal cell shape changes or OM biogenesis defects, and enable bacterial cells to survive under stress conditions (14, 18, 20). In this study, we present the crystal structure of DpaA and structure-based mutational analysis to provide insight into the molecular basis of this unique amidase activity for the detachment of Lpp from PG.

## RESULTS

### Overall structure of DpaA

We initially conducted crystallization trials using recombinant *E. coli* DpaA (UniProt accession number: P0AA99), which contained residues 20-246, lacking the N-terminal signal peptide (hereafter named sDpaA). Unfortunately, attempts to crystallize sDpaA containing either the wild-type catalytic residue Cys143 or the C143A mutation were unsuccessful. However, by treating purified sDpaA with V8 protease (as described in the Materials and Methods), we were able to obtain crystals of the V8-trimmed wild-type sDpaA. Subsequently, we collected a 2.9 Å resolution data set using synchrotron radiation (Table S1).

**Fig 2:**
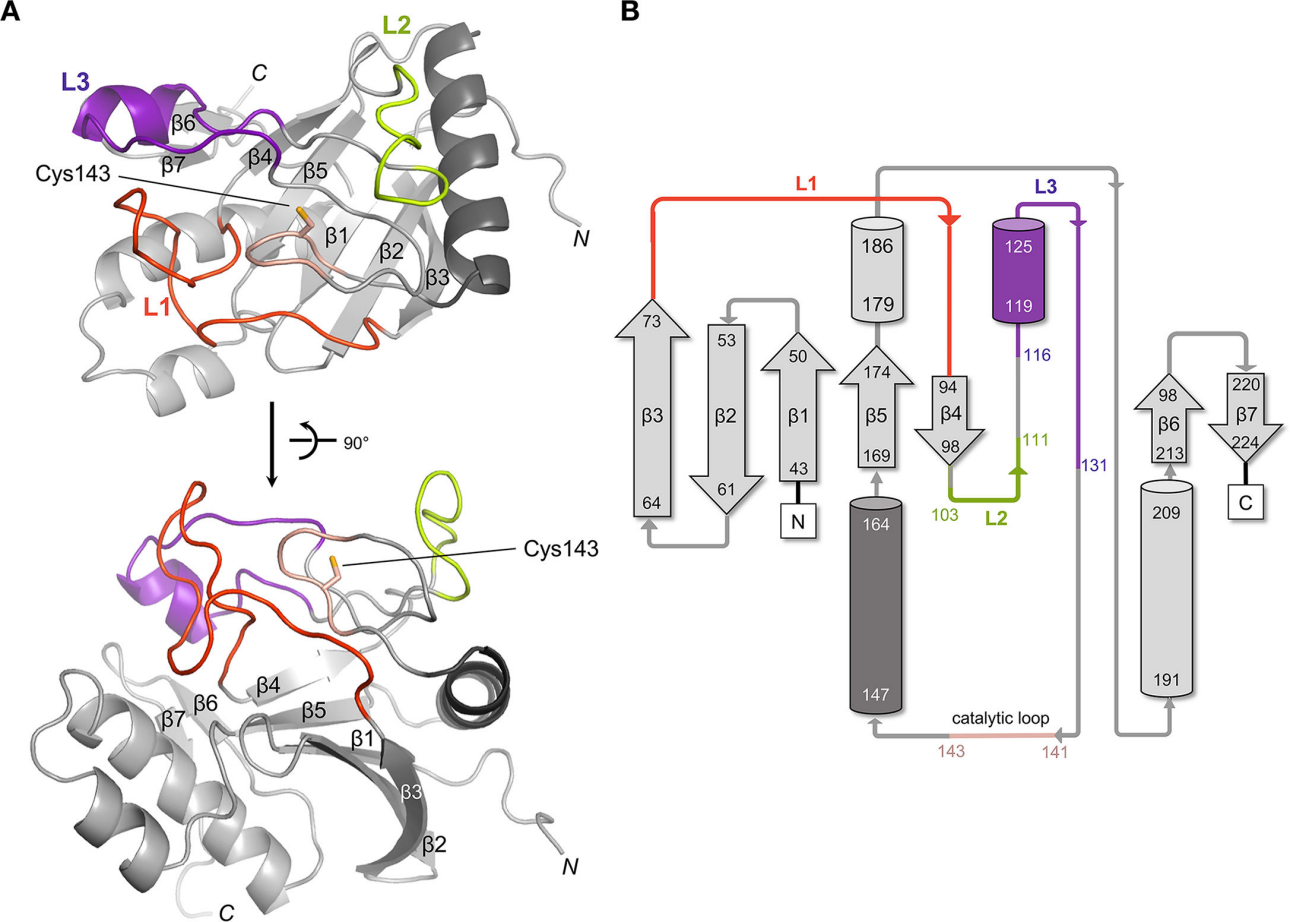
Overall structure of sDpaA. (A) Structure of sDpaA in ribbon representation is shown in two orthogonal views. The loops L1–3 are labeled in orange, green, and purple, respectively. The side chain of the catalytic residue Cys143 is shown in sticks. (B) Secondary structure topology of sDpaA. The starting and ending residue numbers of α-helices (cylinders) and β-strands (arrows) are labeled. The coloring scheme of the structural elements is the same as in (A).

sDpaA forms a pseudosymmetric dimer in the crystal structure. The final model for both chains A and B in the asymmetric unit includes residues 36-229 and 36-224, respectively. Since the chain A model had a lower average B-factor value (82.33 Å^2^) and was more complete, we will refer to chain A in the subsequent discussion. Unlike other YkuD family members, DpaA is a compact protein of a single domain composed of a single mixed five-stranded β-sheet (β1-3 and β4-5), which is flanked by four α-helices and one pair of β-strands (β6-7) (Fig. 2). Unlike other YkuD-like transpeptidases, sDpaA’s second mixed β-sheet region (Val98-Ala145) is degenerated and instead consists of five coiled strands that are connected by only a few backbone hydrogen bonds. The catalytic Cys143 residue is located in a sharp elbow turn of the coils (141-143) and is surrounded by three loops (L1–3) (Fig. 2A and B). These loops create a shallow substrate-binding groove.

By performing a structural similarity search with the crystal structure of sDpaA using the DALI server (21), we found that it shares structural similarities with two LD-TPases, *Bacillus subtilis* YukD (PDB: 1Y7M) and *Mycobacterium tuberculosis* LdtMT2 (PDB: 4GSU), as well as three LD-CPases in the YukD family, *Helicobacter pylori* Csd6 (PDB: 4Y4V), *Bdellovibrio bacteriovorus* Bd1075 (PDB: 7O21), and *Campylobacter jejuni* Pgp2 (PDB: 6XJ6). The *Z*-score, root-mean-square deviation (RMSD), and the sequence identity of these homologous structures are shown in Fig. S1.

### Docking analysis of DpaA with fragments derived from the PG-Lpp substrate

**Fig 3:**
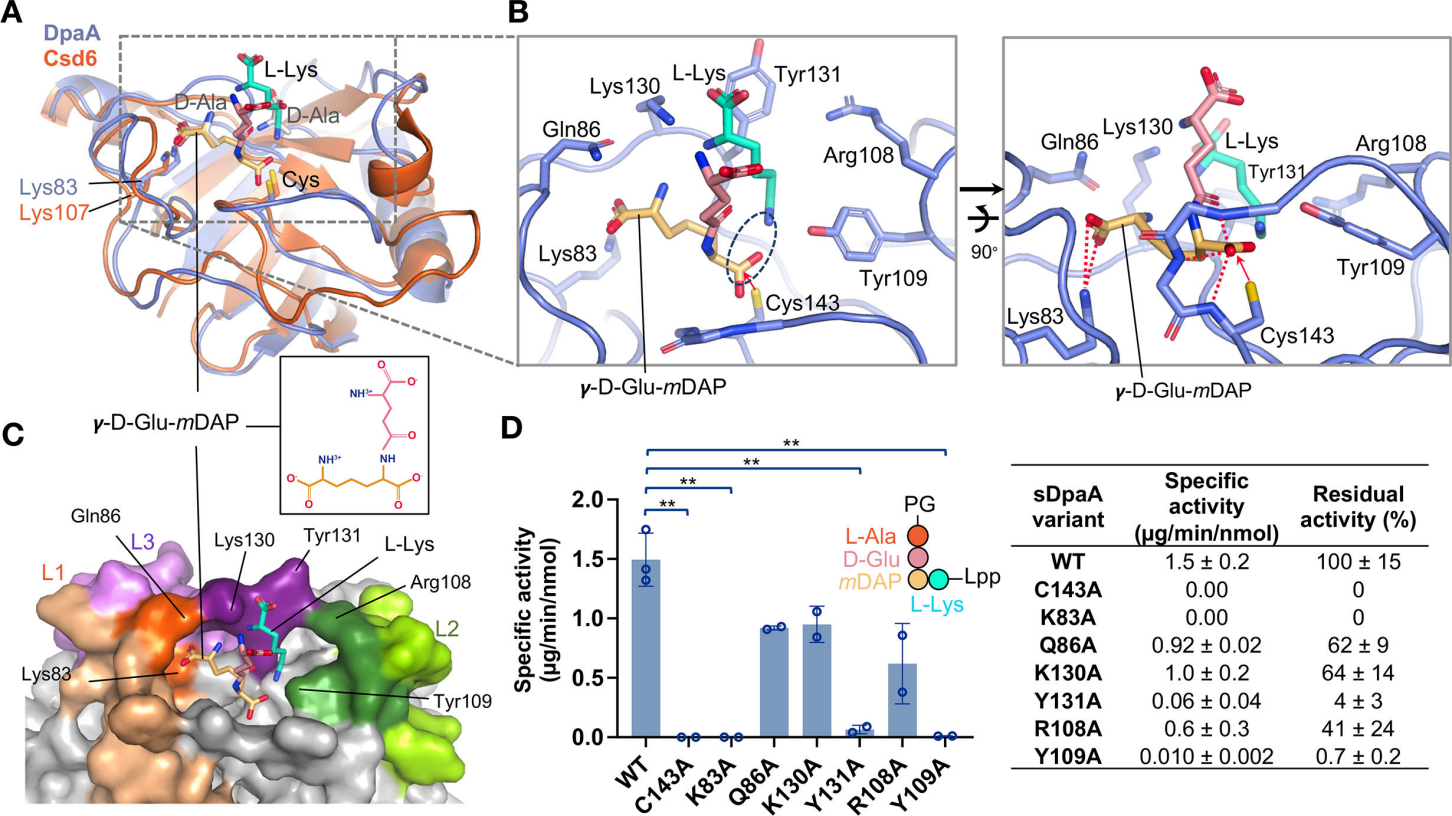
Structure-based mutational analysis and activity assay of DpaA. (A) The stick models of γ-D-Glu-mDAP and L-Lys were manually docked into the active site of sDpaA (blue ribbon model) using the superimposed Csd6-D-Ala structure (PDB code 4Y4V; orange) and the two bound D-Ala residues as guides. The side chains of Lys107 and Lys83 in Csd6 and DpaA, respectively, are shown in sticks, as well as the side chain of catalytic cysteine. The C-terminal tail of DpaA (Lys184-His229) is omitted for clarity. (B) Close-up views of the docking model. The putative substrate-binding residues are shown in sticks. The catalytic Cys143 is colored in yellow. The location of the scissile bond linking γ-D-Glu-mDAP and L-Lys is marked by the dark blue dashed circle. Hydrogen bonds are shown as red dashed lines. (C) sDpaA is shown in surface representation in the same orientation as in (A), with docked stick models of γ-D-Glu-mDAP and L-Lys. The loops L1–3 are labeled in orange, green, and purple, respectively. (D) Bar chart comparing the specific activity of wild-type (WT) sDpaA and the mutants using purified PG-Lpp as the substrate. Unpaired *t*-test was performed to compare the values between groups of the wild-type and mutant proteins (**P value < 0.01). The measured specific activity of WT sDpaA and the mutants is listed in the right table; the values are mean ± standard deviation of at least two independent repeats.

The crystal structure of *H. pylori* Csd6 has been determined in complex with two soaked D-Ala molecules (PDB code 4Y4V); the two D-Ala were thought to occupy the binding sites for the ε-carboxyl side-chain moiety of mDAP and the cleaved product D-Ala (15). The structure of sDpaA does not appear to form an elongated binding groove to accommodate the sugar moiety of the muropeptide or a stem peptide with more than three amino-acid residues. Therefore, we manually docked the models of γ-D-Glu-mDAP and L-Lys, representing the cleaved products, positioned in the catalytic active site of DpaA based on the possible product binding modes suggested by the two D-Ala molecules in 4Y4V (Fig. 3A-C). In our docking model, the scissile amide bond’s carbonyl oxygen is positioned in the oxyanion hole, formed by the catalytic loop residues 141-143, and the scissile carbonyl group is located in close proximity to the side-chain nucleophile of Cys143 (Fig. 3B). The model shows potential substrate-binding residues of sDpaA, exposing a shallow L-shaped binding cleft. Notably, this cleft seems to be able to accommodate only a constrained backbone conformation of an amide bond formed between mDAP and L-Lys, where the stem-peptide backbone of γ-D-Glu-mDAP packs against the long aliphatic side chain of the terminal L-Lys residue of Lpp (Fig. 3B).

The binding site is particularly apparent for the mDAP residue of the substrate, with the shape of the binding groove mainly defined by residues of the L1–3 loops. Specifically, the side-chain amino group of Lys83 from L1 interacts with the carboxylic group at the D-center of mDAP, while Tyr109 from L2 packs against the aliphatic side chain of L-Lys (Fig. 3B), both of which are highly conserved among DpaA-like sequences (Fig. S2). The docked model also highlights four DpaA-specific cleft-lining residues: Gln86 of L1, Arg108 of L2, and Lys130 and Tyr131 of L3. These residues are likely involved in substrate binding. Specifically, Arg108 and Tyr131 may contribute additional packing interactions with the aliphatic side chain of L-Lys in the cleft, while Gln86 may interact with the carboxylic group at the D-center of mDAP (Fig. 3B).

### Structure-based mutational analysis for the amidase activity of DpaA

To test the importance of residues identified by docking analysis, we conducted site-directed mutagenesis by introducing single alanine substitutions for selected residues around the binding cleft, including Lys83 and Gln86 of L1, Arg108 and Tyr109 of L2, and Lys130 and Tyr131 of L3. We assayed the enzyme activity of the sDpaA mutants for cleavage of purified PG with covalently attached Lpp (18) (Fig. 3C). Consistent with our docking model, the K83A mutant exhibited no activity, while the Y109A and Y131A mutants retained only 0.7% and 4% of the wild-type activity, respectively. The mutants Q86A, R108A, and K130A exhibited 62%, 41%, and 64% of the wild-type activity, respectively, suggesting their involvement in substrate binding as well.

Previous studies showed that DpaA is active against the soluble PG muropeptides Tri-LysArg and Tri-Gly, but not Tri-D-Ala (18). Here, we tested the enzyme activity of sDpaA against chemically synthetic compounds mDAP-L-Lys and mDAP-Gly (Supplementary Methods), which contain only the scissile bond between two amino acid residues. Surprisingly, no hydrolysis of these compounds was detected by high performance liquid chromatography (HPLC) analysis after an overnight incubation with sDpaA (Fig. S3). We confirmed this result by conducting isothermal titration calorimetry (ITC) tests for binding of each of the compounds to sDpaA-C143A. The ITC results showed no binding-induced heat change (Fig. S3), indicating that sDpaA does not bind to free mDAP-L-Lys or mDAP-Gly. Taken together, these results suggest that the shallow L-shaped binding cleft of DpaA may only recognize the mDAP-L-Lys or mDAP-Gly amide bonds that are adopted in a constrained conformation, stabilized by hydrogen bonding between the scissile bond residues, L-Lys or Gly, and the stem peptide residues that are missing in the synthetic compounds, discussed below.

## DISCUSSION

### Structural basis for the diverse enzymatic activities of YkuD-like proteins

**Fig 4:**
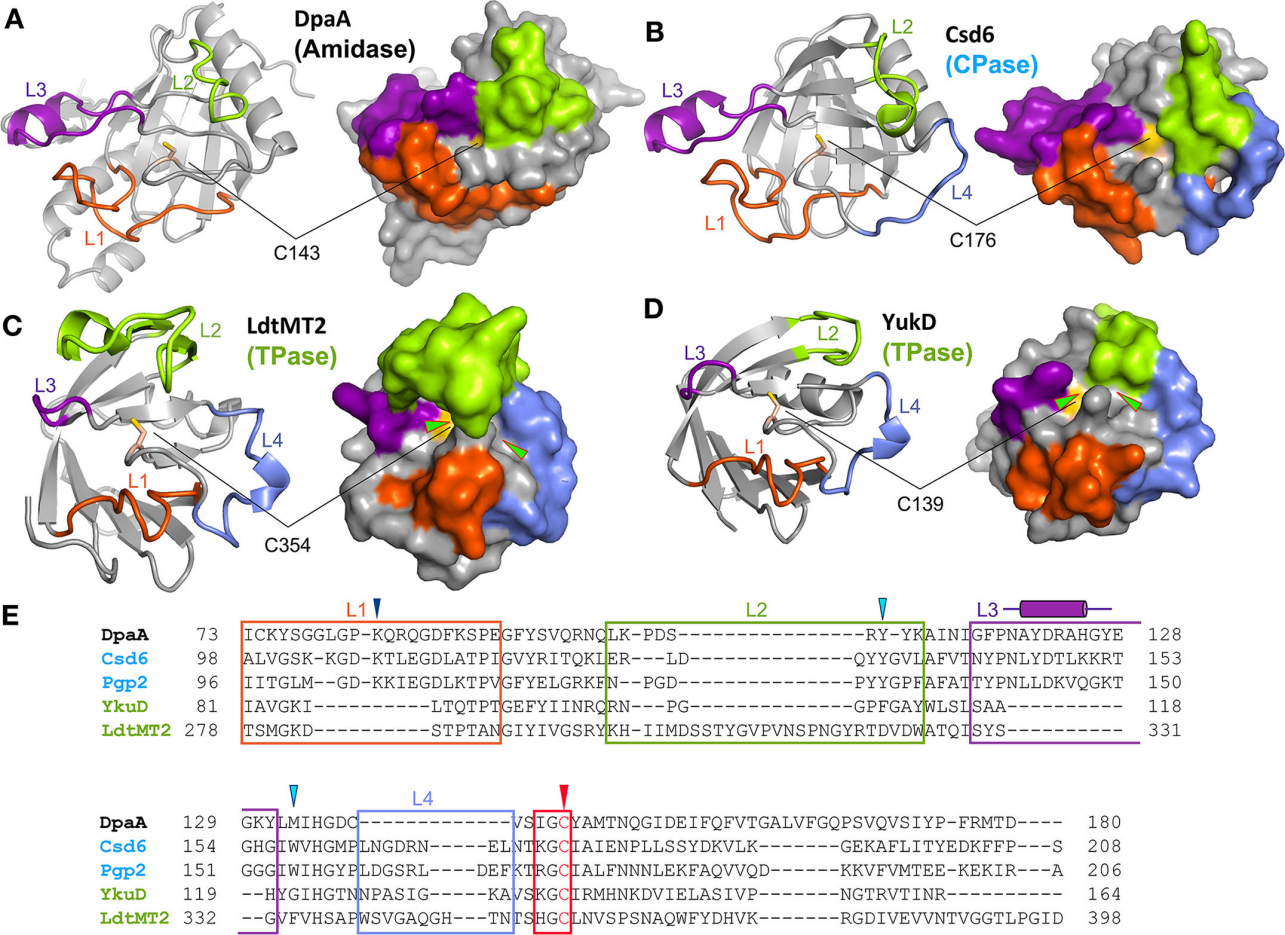
Structural comparison of DpaA with LD-TPases and LD-CPases. (A–D) Cartoon (left) and surface (right) representations of sDpaA (A), the LD-CPase domain of Csd6 from *H. pylori* (B), the LD-TPase domain of LdtMT2 from *M. tuberculosis* (C), and YukD from *B. subtilis* (D). The four loops L1–4 surrounding the catalytic active site are colored and labeled in orange, green, purple, and blue colors, respectively. The catalytic cysteine is shown in yellow. The two active-site entrances on the surface of LD-TPases are marked by arrowheads. (E) Structure-based sequence alignment of sDpaA with two LD-CPases (*H. pylori* Csd6, PDB: 4Y4V; *C. jejuni* Pgp2, PDB: 6XJ6) and two LD-TPases (*B. subtilis* YukD, PDB: 1Y7M; *M. tuberculosis* LdtMT2, PDB: 4GSU). The loop regions are shown in boxes in the same coloring scheme as in (A). The specific residues for DpaA amidase (Lys83) and LD-CPases are labeled with a dark blue triangle. The LD-CPase-specific residues (Tyr132 and Trp158 in Csd6) are indicated by light blue triangles. The catalytic loop containing the active-site residue (Cys143 in DpaA; marked by the red triangle) is shown in the red box.

Our study has revealed that DpaA forms a shallow substrate-binding cleft, with the catalytic Cys143 accessible through a single entrance, similar to LD-CPases (Fig. 4A and B). A pocket-shaped active site is well suited for peptide/amide bond cleavage reactions, as observed in a previous study (15 ). In contrast, the substrate-binding site of DpaA differs from those of the LD-TPases, which have an elongated groove containing the catalytic Cys residue that is accessible via two entrances (Fig. 4C and D), thereby enabling the access of two substrates for peptide-bond formation. An Arg residue, found in many LD-TPases (18 ), is exposed to the elongated groove of the proteins; however, the residue at the equivalent position, Ala145 of DpaA, is buried in the structure.

The substrate-binding grooves of LD-TPases and LD-CPases differ from that of DpaA, as they are formed by four loops separated by β-strands (L1-4) (Fig. 4B-E). In particular, the L1 and L3 loops are considerably longer in LD-CPases and DpaA than in LD-TPases, with the L3 loops in DpaA and LD-CPases containing a helical segment that is absent in LD-TPases. Like in LD-CPases, the L1 and L3 loops in DpaA form a binding pocket for mDAP, which is lined by the conserved Lys 83 playing a critical role in substrate binding (Fig. 3A and B; Fig. 4A; Fig. S2). The L2 loop in LD-TPases forms a lid structure to semi-enclose a substrate-binding groove with two access paths created by the L3 and L4 loops (Fig. 4C and D). By contrast, DpaA and CPases have a shortened L2 loop, resulting in an uncovered substrate-binding site (Fig. 4A and B).

### Substrate binding and selectivity of DpaA

**Fig 5:**
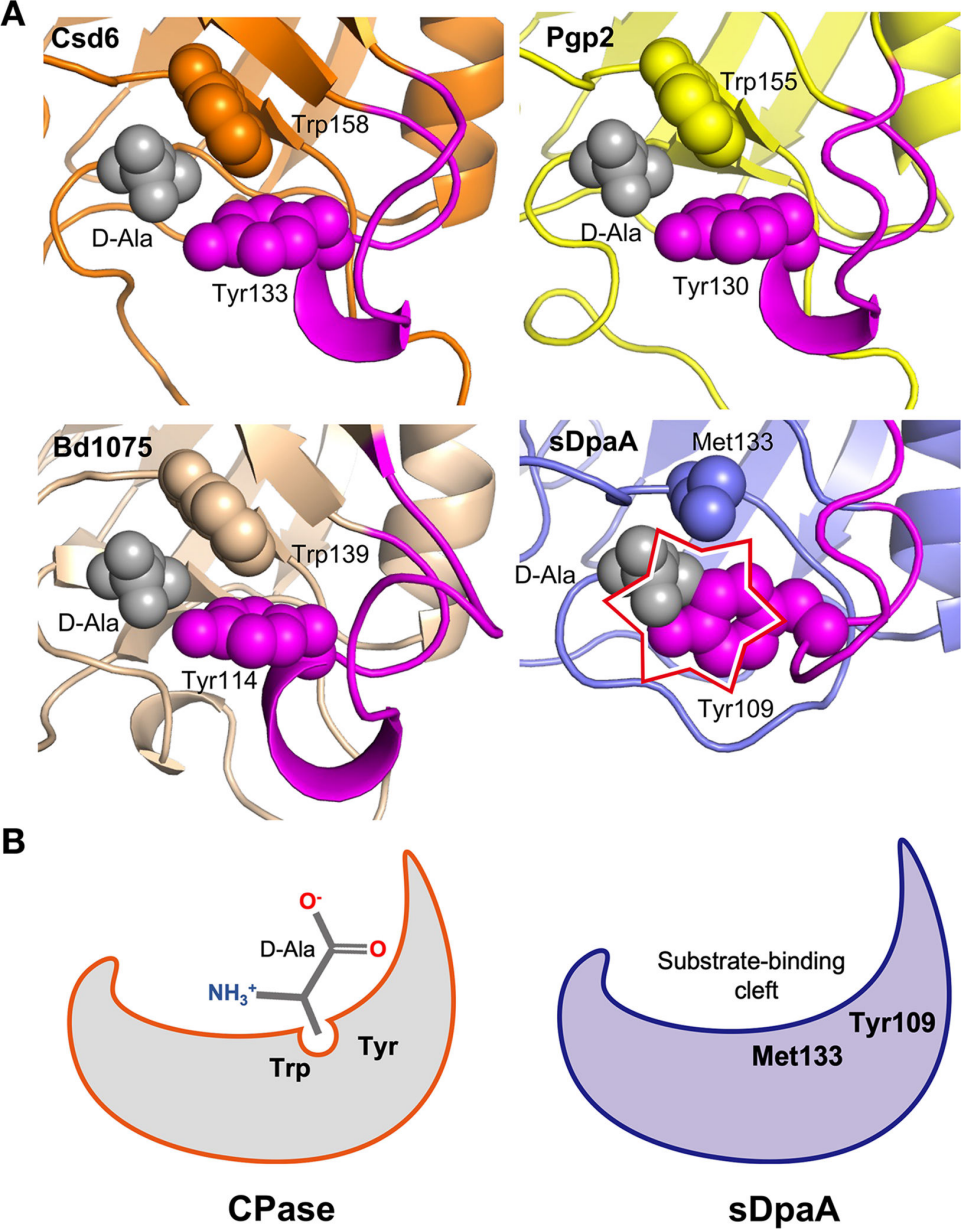
DpaA lacks the LD-CPase-specific D-Ala-binding pocket. (A) The LD-CPase domains of *H. pylori* Csd6 (PDB: 4Y4V, orange), *C. jejuni* Pgp2 (PDB: 6XJ6, yellow), *B. bacteriovorus* Bd1075 (PDB: 7O21, wheat), and sDpaA are aligned and individually shown as ribbon models, with their loop L2 regions colored in magenta. The D-Ala-binding residues, Trp158, and Tyr133, in Csd6 and the corresponding residues in other CPases and DpaA are shown in spheres. The bound D-Ala (gray spheres) in the Csd6 structure is superimposed onto the apo structures of the other CPases and DpaA to show a potential steric clash with the latter, indicated by the star symbol. (B) Cartoon illustration highlighting the structural difference between DpaA and a CPase, the latter possesses a conserved aromatic D-Ala binding pocket, which is missing in the substrate-binding cleft of DpaA.

In addition to its specific PG-Lpp amidase activity, DpaA also functions as a glycine-specific LD-CPase that removes the Gly residue from rare muropeptides with Gly at the fourth position (18, 19). However, unlike the LD-CPase Csd6 (15), DpaA does not cleave muropeptides with D-alanine at the fourth position (18, 19). Structural comparison of the LD-CPase domain of Csd6, Pgp2, and Bd1075 with sDpaA reveals that a D-Ala-binding pocket, lined by two aromatic residues Tyr133 and Trp158 (Fig. 4E), is present in the CPases (Fig. 5A). However, the corresponding residues in DpaA are Tyr109 and Met133, respectively. Moreover, the former Tyr residues are located in the highly variable L2 loops, which adopt different structures in CPases and DpaA (Fig. 4E; Fig. 5A). Consequently, DpaA may not form a binding pocket to accommodate the side-chain methyl group of D-Ala at the fourth position of PG stem peptides (Fig. 5A and B).

**Fig 6:**
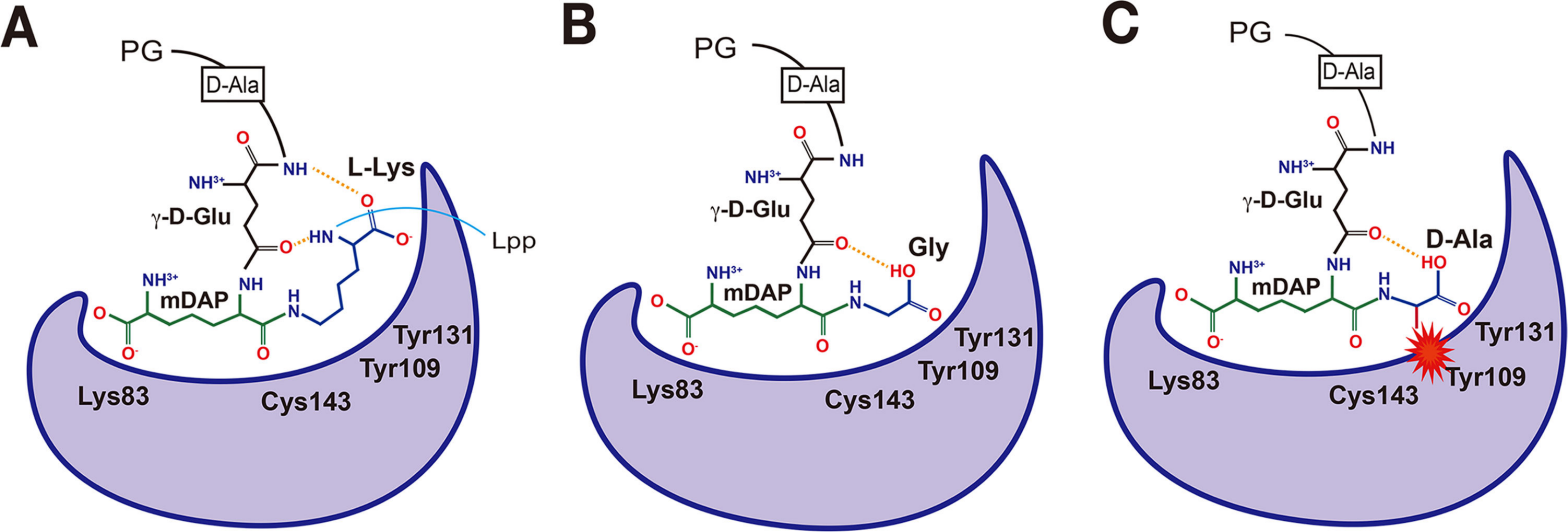
Model of substrate binding and selectivity of DpaA. (A) Cartoon diagram depicting a proposed binding mode of DpaA to the mDAP-L-Lys residues of the stem peptide, which may adopt a constrained conformation induced by intramolecular hydrogen bonds with γ-D-Glu, indicated by dashed lines. (B) Proposed binding modes of DpaA to a stem peptide with Gly at the fourth position. (C) Cartoon diagram illustrating a steric clash between the L2-loop surface region, which contains Tyr109, of DpaA with a stem peptide with the methyl side chain of D-Ala at the fourth position.

Together, these results provide a structural explanation for the substrate selectivity of DpaA (Fig. 6). In this study, we have shown that DpaA cleaves the PG-Lpp substrate, which includes an intact PG tripeptide attached to the C-terminal L-Lys residue of Lpp, but not the derived compound containing only two residues on both sides of the scissile bond. These results suggest that DpaA specifically recognizes the scissile mDAP-L-Lys amide bond that is present in a constrained, rather than extended, conformation. Such constrained conformation of the mDAP-L-Lys linkage may require hydrogen-bonding interactions between the backbone amide and carbonyl groups of L-Lys and the backbone amide and side-chain carbonyl groups of γ-D-Glu in the second position of stem peptide (Fig. 6). Alternatively, the ability of DpaA to cleave the Tri-LysArg muropeptide (to remove the LysArg dipeptide) may point to the presence of further sites in the substrate recognized by DpaA.

## MATERIALS AND METHODS

### Cloning and mutagenesis

Wild-type DpaA containing 20-246 residues (sDpaA), without the periplasmic signal peptide 1-19, was cloned into the pET21b(+) vector with a 6xHis-tag at the C-terminus (19); the plasmid was provided as a gift from Dr. Manjula Reddy (CSIR-CCMB, India). All sDpaA mutants were constructed by site-directed mutagenesis; the primers are listed in Table S2. All constructs were sequenced before use by the DNA Sequencing Core Facility of Academia Sinica (AS-CFII-111-211).

### Protein expression and purification

Cells were grown in LB medium at 37°C until the optical density reached 0.6–0.8. Isopropyl β-D-1-thiogalactopyranoside was then added to a final concentration of 0.5 mM to the culture and incubated overnight at 20°C, allowing expression of sDpaA. Four liters of cells were harvested by centrifugation and suspended in a lysis buffer containing 50 mM Tris-HCl, pH 8.0, and 500 mM NaCl. Cell lysate was ruptured by a French press (Avestin) and centrifuged at 35,000 × *g*. The supernatant was collected and incubated with 2.5 mL of Ni-nitrilotriacetic acid resins (Qiagen) at 4°C for 1 h. The resins were loaded into an open column, washed with 30 mL of lysis buffer and 15 mL of the lysis buffer containing 40 mM imidazole, and then eluted with 15 mL of the buffer containing 250 mM imidazole. The eluted protein was dialyzed overnight against a buffer containing 25 mM HEPES, pH 7.5, 150 mM NaCl, and 1 mM dithiothreitol (DTT). The protein was further purified by a MonoS 5/50 GL column (GE Healthcare) using a gradient of 150–1,000 mM NaCl, followed by size-exclusion chromatography using a Superdex 75 10/300 column (GE Healthcare), equilibrated in 20 mM HEPES, pH 7.5, 300 mM NaCl, 10% glycerol, and 10 mM EDTA.

### Crystallization and data collection

For crystallization experiments, sDpaA was treated with V8 protease (Roche; protein/enzyme ratio, 30:1 wt/wt) at 25°C for 2 h and 4°C overnight, followed by size-exclusion chromatography using a Superdex 75 10/300 column equilibrated in 25 mM HEPES, pH 7.5, 150 mM NaCl, and 1 mM DTT. The purified protein was concentrated to 10–15 mg/mL. sDpaA crystals were grown by the hanging-drop vapor-diffusion method, in solutions containing 19%–25% (wt/vol) PEG 3350, 0.1 M Bis-Tris-HCl, pH 6–7, and 0.2 M ammonium acetate at 22°C. Before data collection, crystals were cryo-protected by transferring to the corresponding reservoir solutions supplemented with 15% ethylene glycol. A data set from a crystal diffracting to 2.9 Å resolution was collected at beamline TLS 15A1 of NSRRC (Taiwan), and was indexed, integrated, and scaled by the XDS program package (22 ).

### Structure determination

Initially, phasing of the diffraction data by molecular replacement using the published structures of Ldt homologues or by anomalous dispersion of Se-Met derivatized crystals was unsuccessful. However, phasing by molecular replacement, using the Ldt-like catalytic domain of Csd6 as the search model (PDB code 4XZZ), combined with Rosetta model improvement in Phenix (23), successfully yielded an electron-density map with sufficient quality for manual model rebuilding. The structure was refined by iterative cycles of manual refitting using Coot (24), and reciprocal-space refinement in Refmac5 (25). Crystallographic and refinement statistics are listed in Table S1.

### ITC

ITC measurements were conducted using a MicroCal iTC200 (Malvern Panalytical, UK) to test the interaction of DpaA-C143A with mDAP-L-Lys or mDAP-Gly. The buffer used for the experiment was 25 mM HEPES, 150 mM NaCl, 0.1 mM tris-(2-carboxyethyl)phosphine (TCEP), pH 7.0, which was filtered using a 0.2 µm filter. The sample cell temperature was maintained at 25°C, the reference power was set to 5 μcal/s, and an initial delay of 120 s was provided with a stirring speed of 750 rpm. All titrations were carried out by injecting 0.4 µL initially, followed by 19 identical injections of 2 µL of 200 µM mDAP-L-Lys or mDAP-Gly into a sample cell containing 20 µM of DpaA-C143A. The time interval between the consecutive injections was fixed at 150 s, and each injection had a duration of 4 s. The heat of dilution obtained from the titration of compound in buffer and buffer in protein were subtracted from each injection. The data were analyzed and plotted using MicroCal PEAQ-ITC software 1.21 (Malvern Panalytical, UK).

### Activity assays

PG with covalently-attached Lpp was isolated as previously described in the standard PG purification method by Glauner, but with the omission of the treatments with α-amylase and pronase E (18, 26). Time-course assays were performed to measure the specific activity of wild-type DpaA and the mutants. Samples contained sDpaA at a concentration of 10 µM for Y109A and K83A or 0.6 µM for the other sDpaA versions and 13.5 µL PG in a total volume of 90 µL. PG and DpaA were diluted separately in reaction buffer (20 mM HEPES, 100 mM NaCl, 0.05% Triton X-100, 0.1 mM TCEP) at twice the final concentration. Reactions were started by mixing equal volumes of the diluted components and incubated at 30°C. Twenty microlitre samples from the mixture were taken after 4, 8, 16, and 30 min, and the reactions were stopped by addition of 1 mM CuCl_2_ and incubation on ice. Samples were then centrifuged for 15 min at 4°C and 13,300 × *g*. The supernatants containing the released Lpp were analyzed together with His-tagged Lpp protein as quantification standard by SDS-PAGE (sodium dodecyl sulfate polyacrylamide gel electrophoresis) followed by Coomassie blue staining. The amount of released Lpp was calculated by densitometry of the gels. The specific activity was calculated with the amount of released Lpp at each time point.

Reverse-phase HPLC was performed to detect the activity of DpaA against mDAP-L-Lys or mDAP-Gly. The powders of mDAP-L-Lys and mDAP-Gly were solubilized in 100 µL distilled water and freeze-dried twice to remove trifluoroacetic acid (TFA). DpaA (240 µM) was incubated with 240 µM mDAP-L-Lys or mDAP-Gly in a total volume of 50 µL buffer containing 25 mM HEPES, pH 7.0, 150 mM NaCl, 5 mM DTT at 37°C, 350 rpm for 14 h. The pH of the sample mixtures was verified using test strips before the incubation. The samples without DpaA, with DpaA only, with buffer only, and with 1.9 mM DAP only were separately injected as controls. Each sample was added with 100 µL of acetonitrile (ACN) and centrifuged for 10 min at 10,000 × *g* before injection into a TSKgel Amide-80 3 µm column (Merck), using the absorbance at 214 nm for detection. Gradient elution programs were used with a flow rate of 1 mL/min. The elution gradient for mDAP-L-Lys started with a mixture of 95% ACN and 5% distilled H_2_O, which was gradually decreased to 5% ACN in 30 min. mDAP-L-Lys was eluted at 13 min while mDAP was eluted at 12 min. For the separation of mDAP-Gly and mDAP, the elution gradient started with 90% ACN and was decreased to 5% ACN in 30 min. mDAP-Gly was eluted at 19.6 min while DAP was eluted at 15.7 min.

## Data Availability

The structural factors and coordinates have been deposited in the Protein Data Bank under the accession code 8IKR. The docking model has been deposited in ModelArchive and is available at https://modelarchive.org/doi/10.5452/ma-y2f7x.

## SUPPLEMENTAL MATERIAL

Fig. S1 mbio.01379-23-s0001.tif Structural homologues of *E. coli* DpaA.

Fig. S2 mbio.01379-23-s0002.tif Sequence alignment of DpaA-like proteins, LD-CPases, and LD-TPases of the YkuD family.

Fig. S3 mbio.01379-23-s0003.tif DpaA is inactive against mDAP-L-Lys and mDAP-Gly.

Legends mbio.01379-23-s0004.pdf Legends for Fig. S1 to S3.

Supplemental Methods mbio.01379-23-s0005.pdf Chemical synthesis of mDAP-Gly and mDAP-L-Lys.

Table S1 mbio.01379-23-s0006.pdf Crystallographic data collection and refinement statistics.

Table S2 mbio.01379-23-s0007.pdf List of primer sequences used in the study.

ASM does not own the copyrights to Supplemental Material that may be linked to, or accessed through, an article. The authors have granted ASM a non-exclusive, world-wide license to publish the Supplemental Material files. Please contact the corresponding author directly for reuse.
