# Supplementary figures and images for: Structural basis for the hydrolytic activity of the transpeptidase-like protein DpaA to detach Braun’s lipoprotein from peptidoglycan
Source: mBio. 2023 Oct 13;14(5):e01379-23. doi: 10.1128/mbio.01379-23 (PMC10653827; doi:10.1128/mbio.01379-23)

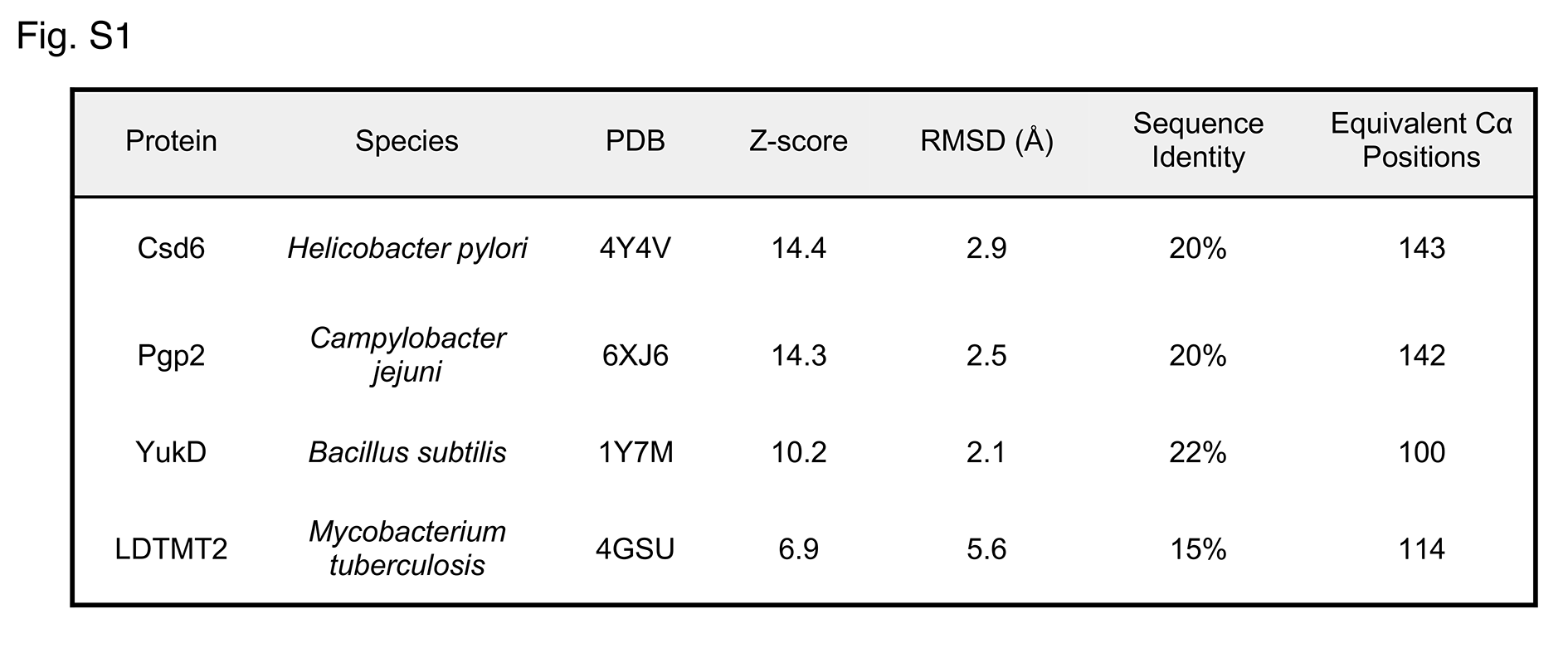

Supplement: Fig. S1 — Structural homologues of E. coli DpaA. [file mbio.01379-23-s0001.tif]

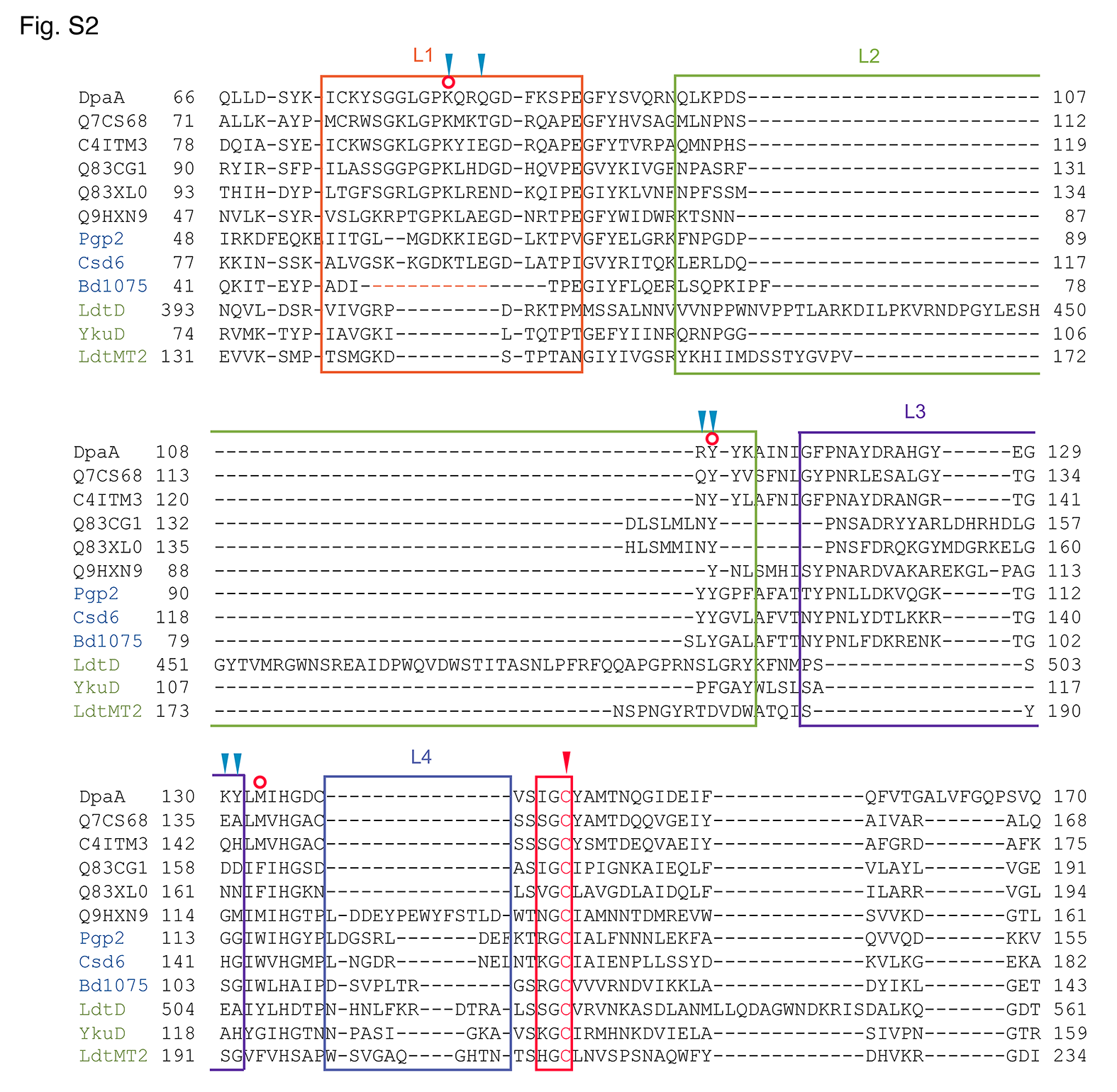

Supplement: Fig. S2 — Sequence alignment of DpaA-like proteins, LD-CPases, and LD-TPases of the YkuD family. [file mbio.01379-23-s0002.tif]

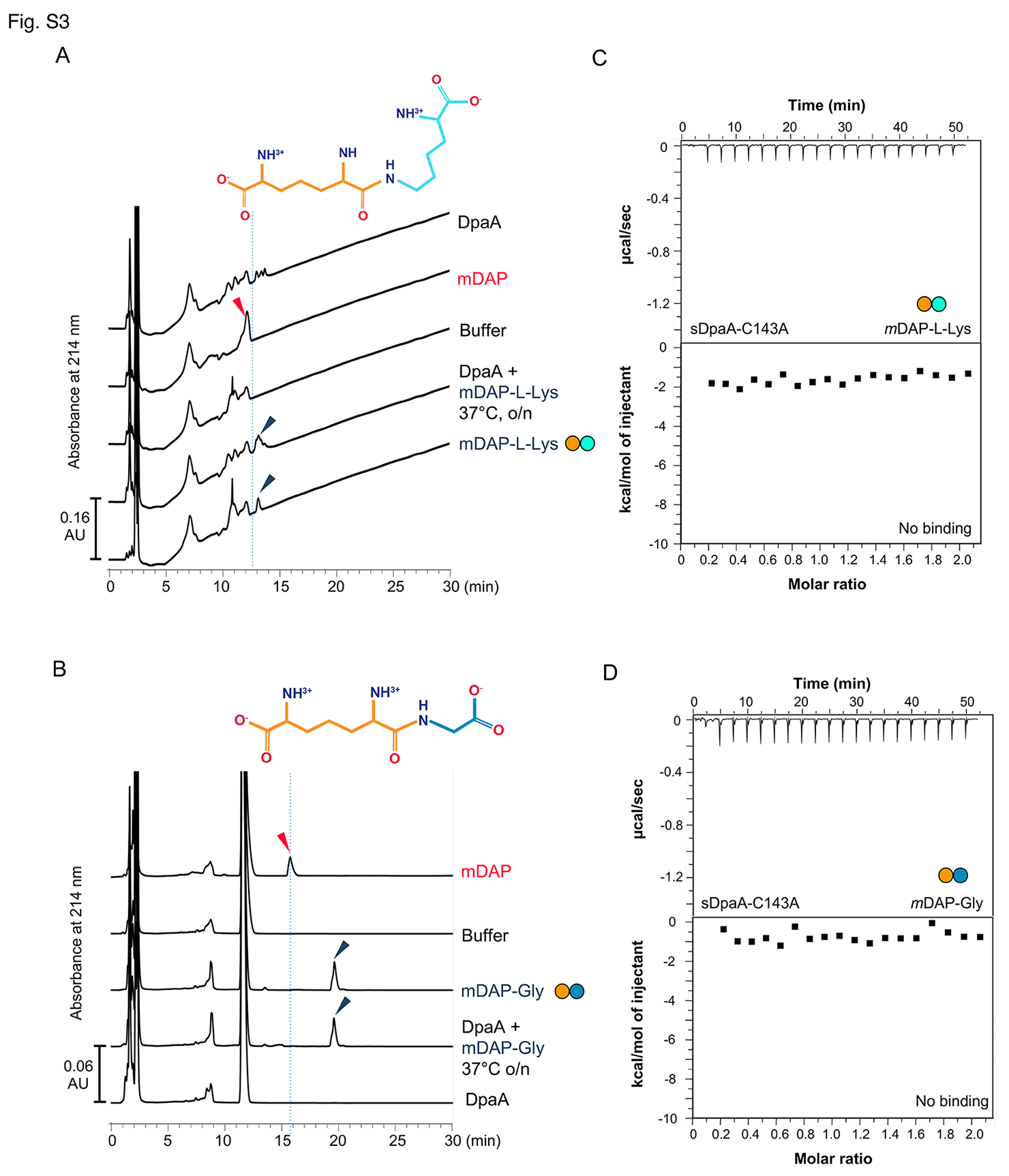

Supplement: Fig. S3 — DpaA is inactive against mDAP-L-Lys and mDAP-Gly. [file mbio.01379-23-s0003.tif]
