# Supplementary material for: Structural basis for the hydrolytic activity of the transpeptidase-like protein DpaA to detach Braun’s lipoprotein from peptidoglycan
Source: mBio. 2023 Oct 13;14(5):e01379-23. doi: 10.1128/mbio.01379-23 (PMC10653827; doi:10.1128/mbio.01379-23)
Supplement: Legends — for Fig. S1 to S3. [file mbio.01379-23-s0004.pdf]

Supplemental Figure Legends:

Figure S1. Structural homologues of *E. coli* DpaA.

Results of structural similarity search using the DALI server. The coordinates of sDpaA were used as the query. The Z score represents the statistical significance of the structural similarity. The RMSD values give the root-mean-square deviation between the corresponding Cα atoms of the target proteins and sDpaA.

Figure S2. Sequence alignment of DpaA-like proteins, LD-CPases, and LD-TPases of the YkuD family.

The alignment was generated using PROMALS3D using the structures of sDpaA, three LD-CPases (*H. pylori* Csd6, *C. jejuni* Pgp2, and *B. bacteriovorus* Bd1075, PDB: 7O21), and three LD-TPases (*E. coli* LdtD, *B. subtilis* YukD, and *M. tuberculosis* LdtMT2) as templates. The residues Gly82-His91 of Bd1075 exhibit poor electron density (indicated by the orange dashed line in loop L1). Therefore, the LD-CPase specific lysine residue can't be located. The sequences of DpaA-like proteins in the YkuD family, including those from *Agrobacterium fabrum* ATU3332, *Brucella abortus* BAB1_2034, *Coxiella burnetii* CBU_1157, *Legionella pneumophila* LPG1514, and *Pseudomonas aeruginosa* PA3756, were labeled with their UniProt accession numbers. The loops L1-4 surrounding the substrate-binding cleft of sDpaA, LD-CPases, and LD-TPases are highlighted in different colors; the catalytic Cys residues of these sequences are indicated by a red triangle. The residues of DpaA that were used in site-directed mutagenesis in this study are labeled with blue triangles. The residues that can be used to distinguish DpaA-like amidases, LD-CPases, and LD-TPases are labeled with red circles.

Figure S3. DpaA is inactive against mDAP-L-Lys and mDAP-Gly.

Activity of DpaA on mDAP-L-Lys (A) and mDAP-Gly (B) was detected by reverse-phase HPLC using different elution programs made for each compound (see Methods). The peaks of mDAP injected as the standard are indicated by the red arrows while the peaks of mDAP-L-Lys or mDAP-Gly are indicated by blue arrows. Isothermal titration calorimetry (ITC) analysis of the binding between sDpaA-C143A and synthetic mDAP-L-Lys (C) or mDAP-Gly (D), showing the raw data (top) and negative binding isotherm derived from the integrated heat (bottom).
