# Supplementary material for: Structural basis for the hydrolytic activity of the transpeptidase-like protein DpaA to detach Braun’s lipoprotein from peptidoglycan
Source: mBio. 2023 Oct 13;14(5):e01379-23. doi: 10.1128/mbio.01379-23 (PMC10653827; doi:10.1128/mbio.01379-23)
Supplement: Supplemental Methods — Chemical synthesis of mDAP-Gly and mDAP-L-Lys. [file mbio.01379-23-s0005.pdf]

Supplemental Methods:

Chemical synthesis of mDAP-Gly and mDAP-L-Lys

All reactions were conducted in oven-dried glassware under nitrogen atmosphere. The reaction products were purified by using column chromatography on silica gel (Geduran Silicagel 60, 0.040-0.063 mm, from Geduran®), Büchi Pure C-850 FlashPrep automated purification machine monitored by ELSD/UV scan detector or reverse phase WATERS HPLC. Anhydrous solvents and moisture-sensitive materials were transferred using an oven-dried syringe or cannula through a rubber septum. (S)-2-amino-3-enoic acid HCl, (R)-2-aminopent-4-enoic acid HCl, H-Gly-O^{t}Bu HCl salt, *N*-Boc-L-Lys-O^{t}Bu and Grubbs catalyst GII (generation II) were purchased from BLD Pharmatech (Shanghai, China) and were used directly as received without further purification.

The compounds were synthesized according to Schemes 1 and 2 as shown below:

Scheme S1. Synthetic route for mDAP-Gly

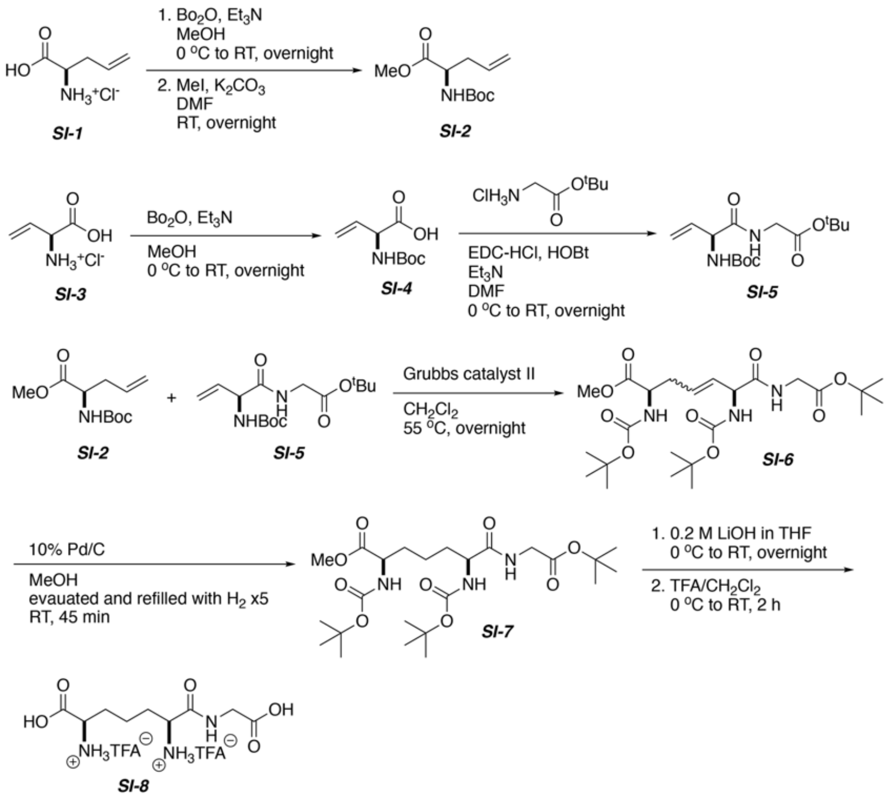

Scheme S2. Synthetic route for mDAP-L-Lys

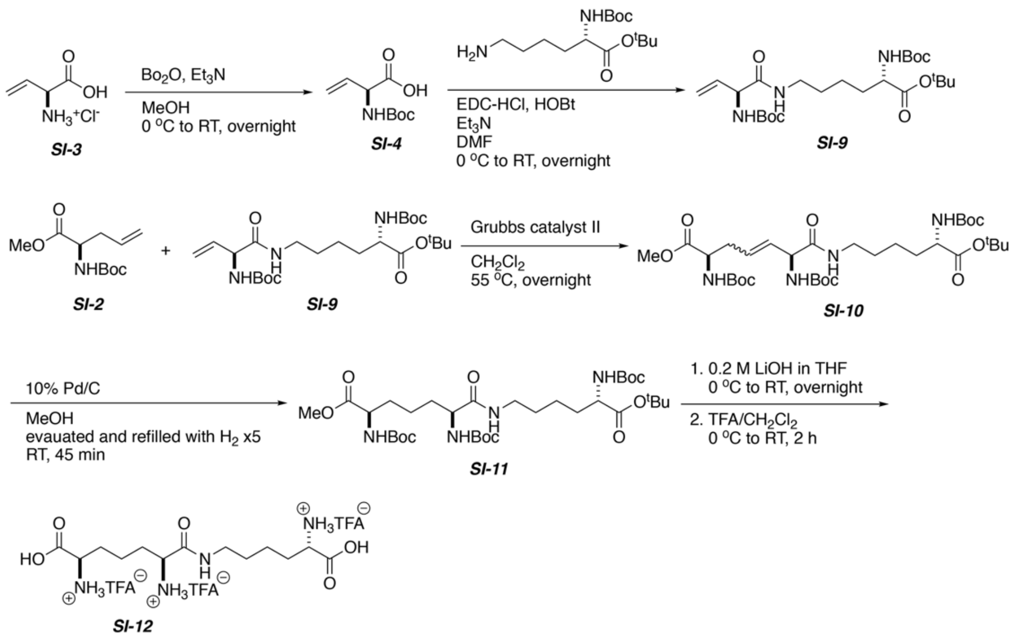

The synthesis of compound **SI-2** follows the standard procedure for the amino and carboxyl group protection on **SI-1**. **SI-5** and **SI-8** were obtained by *N*-Boc-protection of **SI-3** and proceeded to the amide bond formation with the corresponding H-Gly-O^{t}Bu HCl salt, *N*-Boc-L-Lys-O^{t}Bu, respectively, using common coupling reagents. The key steps to get the compounds, mDAP-Gly and mDAP-L-Lys, were to conduct the Grubbs cross metathesis of **SI-2** with **SI-5** and **SI-8**, respectively, followed by hydrogenation and global deprotections: To the solution of **SI-2** (310 mg, 1.36 mmol) and **SI-5** (or **SI-8**) (1.0 equiv.) in dry CH_2Cl_2 (11 mL), Grubbs catalyst GII (10 mol%) was added in one portion with stirring at room temperature under nitrogen atmosphere. The resulting mixture was gently refluxed at 55 °C (oil bath) with stirring overnight (HPLC tracing). CH_2Cl_2 was evaporated and the dryness residue was chromatographically purified on Büchi Pure C-850 FlashPrep automated purification machine monitored by ELSD/UV scan detector to afford the **SI-9** (200 mg, 28% yield) as an amber syrup. The mixture of **SI-9** (40 mg) and 10% Pd/C (20 mg) in dry MeOH was evacuated and refilled with hydrogen gas balloon for 5 times. The reaction

was allowed to stir at room temperature for 45 min (HPLC monitoring), which was filtered through the Millipore hydrophilic PTFE micro-disc (0.45 μm) and concentrated *in vacuo* to give the dryness residue **SI-10**. To the solution of **SI-10** in THF (2 mL), 2 mL of 0.2 M LiOH was added at 0 °C and the resulting mixture was allowed to gradually warm to room temperature with stirring overnight. The solvent was removed *in vacuo* to provide the crude, which was re-dissolved in 2 mL of TFA/CH_2Cl_2 (1/10, v/v) with stirring at room temperature for 2 h. The concentrated crude was passed through the reverse phase WATERS HPLC with the XBridge® Peptide BEH C18 column (5 μm, 4.6x100 mm) at 1 mL/min flow rate under a 30 min gradient of 0-100% solvent B (SolA: H_2O+0.1% TFA, SolB: ACN+0.1% TFA) to afford the desired mDAP-L-Lys (10 mg).

The synthesized compounds were verified by NMR and mass spectrometry.

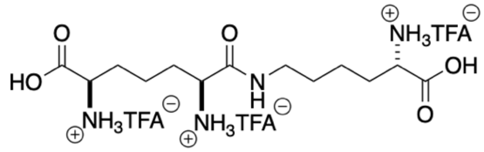

^{1}H NMR (D_2O, 500 MHz) δ 3.97-3.92 (m, 3H, 3xHα), 3.35-3.29 (m, 1H, $CONHCH_2$), 3.27-3.21 (m, 1H, $CONHCH_2$), 1.96-1.88 (m, 6H, 3xCH_2), 1.63-1.43 (m, 6H, 3xCH_2); HRMS (ESI): *m/z* calcd for $C_{13}H_{26}N_4O_5$+H [*M*+H]$^+$ 319.1976; found 319.1983 and [$C_{13}H_{26}N_4O_5$]x2+H [2*M*+H]$^+$ 637.3874; found 637.3911.

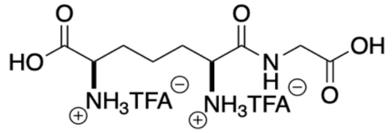

^{1}H NMR (D_2O, 500 MHz) δ 4.09 (d, *J* = 17.1 Hz, 1H, Hα of Gly), 4.08 (t, *J* = 6.3 Hz, 1H, Hα), 4.03 (d, *J* = 17.1 Hz, 1H, Hα of Gly), 3.97 (t, *J* = 6.3 Hz, 1H, Hα), 2.02-1.91 (m, 4H, 2xCH_2), 1.67-1.49 (m, 2H, CH_2); HRMS (ESI): *m/z* calcd for $C_9H_{17}N_3O_5$+H [*M*+H]$^+$ 248.1241; found 248.1243, $C_9H_{17}N_3O_5$+Na [*M*+Na]$^+$ 270.1066; found 270.1063 and [$C_{13}H_{26}N_4O_5$]x2+H [2*M*+H]$^+$ 495.2404; found 495.2417.
