# Supplementary material for: Structural basis for the hydrolytic activity of the transpeptidase-like protein DpaA to detach Braun’s lipoprotein from peptidoglycan
Source: mBio. 2023 Oct 13;14(5):e01379-23. doi: 10.1128/mbio.01379-23 (PMC10653827; doi:10.1128/mbio.01379-23)
Supplement: Table S1 — Crystallographic data collection and refinement statistics. [file mbio.01379-23-s0006.pdf]

Table S1. Crystallographic data collection and refinement statistics

	sDpaA
**Data collection**	
Wavelength (Å)	1.0000
Space group	P 4 2_1 2
Cell dimensions	
a, *b*, *c* (Å)	86.876, 86.876, 150.675
α, β, γ (°)	90, 90, 90
Resolution (Å)	28.47-2.9 (2.974-2.9)[a]
I/σI	26.07 (2.98)
R_{pim}[b]	0.019 (0.406)
$CC_{1/2}$[c]	0.999 (0.888)
Completeness (%)	99.49 (98.99)
Redundancy	2.0 (2.0)
**Refinement**	
Resolution (Å)	28.47-2.9 (2.974-2.9)
No. of reflections	12702 (861)
Reflections used for R_{free}	661 (50)
R_{work}	0.21461 (0.364)
R_{free}	0.27370 (0.348)
No. atoms	
Protein	3033
B-factors (Å^2)	
Protein	111.576
R.m.s. deviations	
Bond lengths (Å)	0.01
Bond angles (º)	1.855
Validation	
Clash score	9.69
Rotamer outliers (%)	8.54
Ramachandran plot	
Favored/Allowed/Disallowed (%)	88.89/11.11/0

[a] Highest resolution shell is shown in parenthesis.

[b] R_{pim} is the precision-indicating merging *R*, which describes the accuracy of the averaged measurement (1).

[c] $CC^{1/2}$ is the correlation coefficient between two random half data sets (2).

References:

1. Weiss MS. 2001. Global indicators of X-ray data quality. Journal of Applied Crystallography.
2. Karplus PA, Diederichs K. 2012. Linking crystallographic model and data quality. Science 336:1030–1033.
