# Supplementary material for: Structural basis for the hydrolytic activity of the transpeptidase-like protein DpaA to detach Braun’s lipoprotein from peptidoglycan
Source: mBio. 2023 Oct 13;14(5):e01379-23. doi: 10.1128/mbio.01379-23 (PMC10653827; doi:10.1128/mbio.01379-23)
Supplement: Table S2 — List of primer sequences used in the study. [file mbio.01379-23-s0007.pdf]

Table S2. List of primer sequences used in the study

K83A forward	TATTCCGGCGGCTTAGGGCCAGCACAGCGTCAGG
K83A reverse	CCTGACGCTGTGCTGGCCCTAAGCCGCCGGAATA
Q86A forward	GGGCCAAAACAGCGTGCGGGCGATTTCAAAAG
Q86A reverse	CTTTTGAAATCGCCCGCACGCTGTTTTGGCCC
R108A forward	CAGACAGCGCGTACTACAAAGCGATTAATATTG
R108A reverse	TTTGTAGTACGCGCTGTCTGGTTTTAACTGATT
Y109A forward	CAGCCGTGCGTACAAAGCGATTAATATTGGTTTC
Y109A reverse	CTTTGTACGCACGGCTGTCTGGTTTTAACTG
K130A forward	CATGGTTATGAAGGGGCATACCTGATGATCC
K130A reverse	GGATCATCAGGTATGCCCCTTCATAACCATG
Y131A forward	GTTATGAAGGGAAAGCCCTGATGATCCACG
Y131A reverse	CGTGGATCATCAGGGCTTTCCCTTCATAAC
C143A forward	GATTGTGTTTCCATCGGC GCA TACGCAATGACCAATCAG
C143A reverse	CTGATTGGTCATTGCGTA TGC GCCGATGGAAACACAATC
